# Maternal and Perinatal Outcome in a Contemporary Cohort of Patients with Portal Hypertension: A Single-Center Experience

**DOI:** 10.3390/jcm12093088

**Published:** 2023-04-24

**Authors:** Philipp Kosian, Christian Jansen, Johannes Chang, Michael Praktiknjo, Tiyasha Hosne Ayub, Ulrich Gembruch, Waltraut M. Merz

**Affiliations:** 1 Department of Obstetrics and Prenatal Medicine, University Hospital Bonn, Venusberg Campus 1, 53127 Bonn, Germany; 2 Department of Internal Medicine I, University Hospital Bonn, Venusberg Campus 1, 53127 Bonn, Germany

**Keywords:** portal hypertension, pregnancy, maternal complication, variceal bleeding

## Abstract

Background: Portal hypertension in pregnancy is characterized by an increased perinatal and maternal complication rate. The purpose of this study was to evaluate the perinatal and maternal outcomes of these high-risk pregnancies at our tertiary center. Methods: We identified pregnancies with portal hypertension in our departmental database for the years 2013 to 2021. The medical history and perinatal and maternal data were extracted from medical records. Results: Eleven cases were identified. In pregnancy, delivery and postpartum, complications occurred in 72.7% of cases and included among others ascites, subclavian thrombosis, variceal-ligation-induced ulcer bleeding and postoperative hemorrhage. The cesarean delivery rate was 72.7% (*n* = 8); five of these were done for obstetric or fetal indications. The rate of preterm birth and admissions to neonatal intensive care unit were high (54.5% and 45.5%, respectively). Conclusions: Our case series substantiates the high maternal and perinatal complication rates seen in portal hypertension. The prevention of thromboembolic and bleeding complications was the main challenge. Care by an interdisciplinary team of experts is crucial for a successful perinatal and maternal outcome.

## 1. Introduction

Portal hypertension is the result of increased vascular resistance in the portal circulation and increased portal venous blood flow. It is defined by the portosystemic pressure gradient (PSPG). Values of more than 10 mmHg are defined as clinically significant portal hypertension (CSPH) [1]. Portal hypertension may develop in patients with cirrhosis, as well as in patients without cirrhosis in the case of vascular disorders of the liver. Complications of portal hypertension include variceal bleeding, ascites, hypersplenism and hepatic encephalopathy [1].

Hemodynamic changes associated with pregnancy include an increase in blood volume and cardiac output and a decrease in systemic vascular resistance. Therefore, an increase in portal blood flow and portal pressure can be observed. This consecutively higher portal pressure in collateral veins increases the risk of variceal bleeding in this group of patients [2]. Pregnancy-associated hypercoagulability, on the other hand, may increase the risk of thromboembolic complications. Pregnancies in women with portal hypertension are therefore characterized by higher maternal and perinatal complication rates. Historically, up to 24% of cirrhotic patients with portal hypertension were reported to experience variceal bleeding at some point during pregnancy [3]. More recently published population-based studies describe a risk of around 5% [4,5]. In a recent series of 45 non-cirrhotic patients with portal hypertension, data revealed a risk of 6.6%, whereas historic data reported a risk of approximately 15% [6,7]. A systematic review and meta-analysis describing maternal and perinatal outcomes in portal hypertension in 581 patients (895 pregnancies) observed 22 maternal deaths mostly after variceal bleeding and hepatic decompensation. Variceal bleeding occurred in 14% of patients [8]. An increased risk for cesarean delivery and preterm birth is known, especially in patients with cirrhosis and portal hypertension [4,9,10,11,12,13,14,15]. In patients with liver cirrhosis, the estrogen and endocrine metabolism is affected, leading to anovulation and amenorrhea [10]. Therefore, pregnancies in patients with portal hypertension, particularly in those with cirrhosis, are still rare, but as a result of improvements in care, pregnancy rates in this group of patients are increasing [4]. With a high risk for complications and limited published data available, the purpose of this study was to evaluate the perinatal and maternal outcomes of these high-risk pregnancies at our tertiary center, a liver transplant center with an annual outpatient pregnancy volume of around 3000 patients.

## 2. Materials and Methods

For the years 2013 to 2021, the terms “portal hypertension”, “esophageal varices”, “Budd-Chiari”, “cirrhosis”, “portal vein thrombosis”, “hepatitis” and “ascites” were searched in our departmental obstetric database; here, all women with portal hypertension were included, and their medical history and perinatal and maternal data were extracted from medical records.

The patients’ notes were then retrieved for details of their medical and obstetric history, course of pregnancy, delivery and maternal and perinatal outcome. Relevant maternal details (maternal age, etiology of portal hypertension, number of esophagogastroduodenoscopies (EGD) in pregnancy, albumin–bilirubin score (ALBI score), presence of varices and/or thrombocytopenia), pregnancy outcomes, months of follow-up and condition at the last follow-up were evaluated (Tables S1 and S2 in the Supplementary Materials). Outcomes including gestational age at delivery, mode of delivery, rate of complication, blood loss, presence of thrombocytopenia and presence of varices were compared in patients with cirrhotic and non-cirrhotic portal hypertension (Table 1). Statistical analysis was performed using IBM SPSS statistics, version 27 (SPSS Inc., an IBM Company, Chicago, IL, USA). Mann–Whitney U test and Fisher’s exact test were used for univariate analysis wherever appropriate. *p*-value < 0.05 was considered significant. The study was conducted in accordance with the Declaration of Helsinki. Ethical review and approval were waived in view of the retrospective nature of the study by the Ethics Committee of the Medical Faculty of the University of Bonn.

## 3. Results

A total of 11 cases out of 18,589 patients (0.6‰) giving birth at our center between 2013 and 2021 were identified. Tables S1 and S2 (found in the Supplementary Materials) summarize our findings related to these women. The mean age was 32 years (SD 7.37; range 20–44). All patients were referred to our center for management and had an established diagnosis of portal hypertension either by balloon wedge pressure measurement (*n* = 1) or by clinical manifestation (ascites, varices, variceal bleeding or splenomegaly) and ultrasound. Six out of eleven patients were cared for at the Department of Internal Medicine, University of Bonn, prior to pregnancy. The other patients were first referrals at various stages of pregnancy. No patient had undergone preconception counseling by a maternal–fetal medicine specialist. The etiology was cirrhosis in 45.4% (*n* = 5) of cases; in three of those, the cause was alcoholic liver cirrhosis. In 36.4% (*n* = 4), Budd Chiari (in two cases with fibrosis), and in 18.2% (*n* = 2), non-cirrhotic portal vein thrombosis was the underlying cause. The majority women (10/11) had experienced complications of the underlying condition prior to pregnancy (Table S1). In total, 15 EGD were performed either before pregnancy (4 cases), or in the first (1 case), second (3 cases), or third trimester (7 cases). Two women underwent variceal banding, one in the second and one in the third trimester. Apart from one EGD performed due to variceal-ligation-induced ulcer bleeding, EGDs were performed electively in view of the underlying disease and the presence of pregnancy, which is considered an additional risk factor. Four of the nine patients with varices received prophylaxis with a beta blocker (carvedilol or propranolol). One patient (case 10) was recommended to take beta blockers but was non-compliant, and one patient (case 4) discontinued the medication due to side effects.

Maternal complications during pregnancy, delivery and puerperium occurred in 36.4% (4/11) of cases and included, among others, variceal-ligation-induced bleeding (case 10), ascites (case 1 and 5), postoperative hemorrhage (case 1) and subclavian thrombosis (case 5). Six of the eleven patients were on anticoagulation medication, including the patients that suffered postoperative hemorrhage (case 1) and thrombosis (case 5) (Table S2). For the patients on prophylactic anticoagulation, medication was paused for delivery, whereas patients on therapeutic anticoagulation were bridged with heparin. Additional pre-existing medical conditions are documented in Table S1.

Obstetric complications in our series occurred in 7/11 cases and included gestational diabetes (*n* = 3), fetal growth restriction (*n* = 1), preeclampsia (*n* = 1), abnormal fetal Doppler indices (*n* = 1), intrahepatic cholestasis of pregnancy (ICP) (*n* = 2) and one case of preterm premature rupture of membranes, resulting in preterm delivery at 31 + 6 weeks of gestation. In 3/8 of cases, pregnancy-related and maternal complications correlated and occurred together.

The gestational age (GA) at birth ranged from 28 to 40 weeks (median 35 weeks of gestation, IQR 5). The cesarean delivery rate was 72.7% (*n* = 8); five of these were performed for obstetric or fetal indications. The preterm birth and neonatal intensive care unit admission rate was high (54.5% and 45.5%, respectively). Birthweight was appropriate for gestational age (median percentile 32, IQR 40). Umbilical arterial pH values ranged from 7.07 to 7.36 (median 7.32, IQR 0.08). The 5 min Apgar score was ≥7 in 90.9% (10/11) of cases.

One life-threatening acute variceal-ligation-induced ulcer bleeding occurred in pregnancy week 33 + 5, resulting in an emergency cesarean delivery with simultaneous injection of fibrin tissue glue and variceal banding distal of the bleeding ulcus. The estimated blood loss due to the variceal bleeding was 2000 mL and the intraoperative blood loss was 500 mL, adding up to an estimated total blood loss of 2500 mL. The patient had a history of alcoholic liver cirrhosis Child–Pugh stage C and had undergone ligation of esophageal varices grade III twelve days prior to the event.

During follow up, we observed two cases of death. One patient with Budd Chiari syndrome died of hepatic decompensation after a failed liver transplantation 19 months after delivery (case 5), and a second patient died 30 months after delivery, presenting with hypovolemic shock due to retroperitoneal hematoma of unknown etiology (case 9).

We observed no difference in the occurrence of varices (*p* = 0.99), gestational age at birth (*p* = 0.43), blood loss (*p* = 0.43), mode of delivery or rate of complication (*p* = 0.99) between patients with cirrhotic compared to non-cirrhotic portal hypertension. Thrombocytopenia was more common in women with cirrhosis, but this did not reach statistical significance (*p* = 0.08) (Table 1).

All patients were cared for by an interdisciplinary team of experts in obstetrics, prenatal medicine, hepatology, hematology, anesthesia and intensive care.

## 4. Discussion

Our case series confirms the high maternal and perinatal complication rates associated with portal hypertension.

A prospective study analyzing the outcome of 165 pregnancies with chronic liver disease found a higher rate of stable disease in patients who had preconception counseling. In our series, no patient received preconception counseling; this fact may have contributed to the high rate of maternal complications [16].

In four case series describing pregnancies in women with portal hypertension caused by cirrhosis, variceal bleeding occurred in 3% to 42% of the cases [11]. In our series, we reported one episode of variceal-ligation-induced ulcer bleeding in a patient with prior decompensated liver cirrhosis (variceal bleeding and ascites). In the data published by Flemming et al., 2020, women with decompensated cirrhosis were more likely to have ICP, preterm birth, cesarean delivery and small-for-gestational-age newborns compared to patients without prior decompensation. In our case series, 3/5 patients with cirrhosis and portal hypertension had decompensated liver cirrhosis before pregnancy, which is a higher rate compared to the study by Flemming et al. [12]. We also observed a high rate of preterm birth in women with portal hypertension and cirrhosis (4/5 cases, median gestational age 35.0, IQR 2.5) and an increased rate of cesarean delivery (80%; 4/5 cases). ICP was observed in 2/5, and small-for-gestational-age newborns in 1/5 cirrhotic patients with portal hypertension. Live birth rates in pregnancies of women with cirrhosis are reported between 58% and 100% [15]. In our series, we had a live birth rate of 100%. An ALBI score grade 1 is associated with a positive outcome [16]. Only 2/5 of our cirrhotic patients had an ALBI score grade 1.

In women with non-cirrhotic portal hypertension, variceal bleeding is observed in 6.9% to 34% of pregnancies. The data on the preterm birth, gestational age, cesarean delivery and hemorrhage vary widely [11,13,17,18,19]. In our six cases of non-cirrhotic portal hypertension, preterm birth occurred in two cases, and cesarean delivery was performed in four of the six cases. We observed one case of excessive postoperative hemorrhage after CD (case 1).

According to the literature, maternal and perinatal complications are higher in cirrhotic portal hypertension [11]. In our series, we could not detect this difference in outcomes, possibly due to the small number of patients.

The major perinatal complication in our case series overall was prematurity. Interdisciplinary care by a team of experts seems to be crucial for a successful perinatal and maternal outcome. “Prevention of bleeding and thromboembolic complications turned out to be the major challenge. Factors contributing to the high rate of this type of complications consist of (a) the presence of altered hemostasis secondary to the underlying condition; (b) the need for anticoagulation; (c) pregnancy-induced changes which include a decrease in platelet count, a procoagulatory state, an increased blood volume, a decreased peripheral vascular resistance, and a rise in the intraabdominal pressure.” [20].

Based on our experience and the available literature, we recommend the preconception counseling of patients with known portal hypertension and a baseline evaluation for risk assessment. Female patients of a reproductive age should be counseled early on fertility and pregnancy risks [21]. All patients with known portal hypertension should have a screening endoscopy for varices during the second trimester. Furthermore, prophylaxis with beta blockers should be continued during pregnancy, as benefits outweigh the risks. An interdisciplinary emergency plan including obstetricians, pediatricians and internal medicine specialists should be established to achieve the best outcome in emergency situations.

The limitation of our study is the small number of patients due to the rarity of the condition.

## 5. Conclusions

In conclusion, pregnancies with portal hypertension have a very high maternal morbidity, and these women should be referred to a tertiary center for care during pregnancy. A registry to collect data on portal hypertension in pregnancy may help to identify components of care which contribute to an improved maternal and fetal outcome.

## Figures and Tables

**Table 1:** Outcomes in patients with cirrhotic compared to noncirrhotic portal hypertension. A. *p*-value < 0.05 was considered significant. Abbreviations: CD: cesarean delivery; NVD: normal vaginal delivery.

Baseline Characteristics and Outcomes	Cirrhotic Portal Hypertension (*n* = 5)	Non-Cirrhotic Portal Hypertension (*n* = 6)	*p*-Value
Median thrombocytes G/L	67	161	0.08
Presence of varices (*n*)	4	5	0.99
Median GA at birth (weeks of gestation)	35	37.5	0.43
Median blood loss (mL)	900	450	0.43
Mode of delivery	4 CD, 1 NVD	4 CD, 1 NVD, 1 instrumental delivery	
Cases with complications (*n*)	4	4	0.99

## Data Availability

The datasets used and/or analyzed during the current study are available from the corresponding author on reasonable request.

## Supplementary Materials

The following supporting information can be downloaded at: https://www.mdpi.com/article/10.3390/jcm12093088/s1, Table S1: Maternal baseline variables in eleven cases of portal hypertension in pregnancy. Table S2: Maternal and perinatal outcomes in eleven cases of portal hypertension in pregnancy.
