# Supplementary material for: Maternal and Perinatal Outcome in a Contemporary Cohort of Patients with Portal Hypertension: A Single-Center Experience
Source: J Clin Med. 2023 Apr 24;12(9):3088. doi: 10.3390/jcm12093088 (PMC10179582; doi:10.3390/jcm12093088)
Supplement: Supplementary file 1 [file jcm-12-03088-s001.zip › jcm-2239107-supplementary.pdf]

Case	Age	G	P	Etiology/Underlying cause	Child Pugh Score/ALBI Score	Prior complications	Varices	Platelet count	Medication
1	38	4	3	Fascioliasis, schistosomiasis, portal vein thrombosis; ID 2018	A/Grade 2	Cholestasis, variceal bleeding, splenectomy	Grade I	Normal	Rivaroxaban/Carvedilol
2	20	2	1	Budd Chiari (nicotin/pregnancy); ID 2016	A/Grade 1	Splenic infarction	Gastric varices	Normal	LMWH
3	27	1	0	Budd Chiari (May-Thurner Syndrom); ID 2018	A/Grade 1	LCIV thrombosis	Unknown	119	LMWH
4	30	1	0	Budd Chiari (COC, heterozygous FVL); ID 2011	A/Grade 1	Pulmonary embolism	Grade III	123	LMWH
5	38	3	0	Budd Chiari (COC, thrombocythemia) ID 1999	A/Grade 1	Ascites, TIPS	Grade II/Gastric varices	Normal	LMWH, Fondaparinux
6	27	1	0	Portal vein thrombosis; ID 2000	A/Grade 1	Splenomegaly	Grade III	85	LMWH, Propranolol
7	44	1	0	Cirrhosis (AIH); ID 1998	A/Grade 2	Splenic artery aneurysm	Grade I	24	Azathioprin, Prednisolone, Propranolol
8	24	1	0	Cirrhosis (AIH); ID 2014	n.a.	None	Grade I	102	Prednisolone
9	38	2	1	Cirrhosis (alcoholic); ID 2016	C/Grade 1	Hydropic decompensation, variceal bleeding (rectal)	Grade I	50	Propranolol
10	37	4	2	Cirrhosis (alcoholic); ID 2015	C/Grade 2	Hydropic decompensation, variceal bleeding	Grade III	67	None
11	30	3	1	Cirrhosis (alcoholic); ID 2012	A/Grade 1	Variceal bleeding	None	Normal	Clonazepam, Levomethadon

Table S1 – Maternal baseline variables in eleven cases of portal hypertension in pregnancy. Abbreviations: AIH: autoimmune hepatitis; ALBI score: albumin–bilirubin score; COC: combined oral contraceptive; FVL: Factor V Leiden heterozygous; ID: initial diagnosis; LMWH: low-molecular-weight heparin; n.a.: not available

Case	GA at delivery	Mode of delivery	Indication	Blood loss (mL)	Complications	Newborn weight (percentile)	NICU	Indication for NICU admission	Follow- up in months	Maternal outcome at last follow- up
1	31	CD	PPROM, underlying disease	2200	Ascites, postoperative hemorrhage	75	yes	Respiratory failure	15	Biliary sepsis 11 months post-partum
2	37	NVD (IoL)	Underlying disease	400	None	24	no		27	Stable disease
3	38	CD	Underlying disease	500	None	32	yes	Respiratory failure	9	2 months post-partum iliac stent thrombosis
4	39	CD	Underlying disease	800	GA 22 variceal ligation	31	no		48	Stable disease
5	28	CD	Abnormal NST and Doppler	400	Subclavian thrombosis, TIPS occlusion, ascites, newly occurred varices	64	yes	RDS, neonatal cardiac failure, infection	19	Death 19 months post-partum
6	40	Instrumental delivery	Underlying disease	300	None	35	no		65	Stable disease
7	34	CD	Abnormal fetal Doppler	900	ICP	24	yes	Respiratory failure, Infection, hypotension	36	Stable disease
8	35	CD	Abnormal NST	500	ICP	73	no		95	Stable disease
9	35	CD	Preeclampsia	900	Preeclampsia	11	no		30	Death 30 months post-partum
10	33	Emergency CD	Variceal bleeding	2500	variceal ligation induced ulcer bleeding	41	yes	RDS, sepsis	8	Stable disease
11	37	NVD (IoL)	Underlying disease	150	None	9	no		8	Relapse (alcohol use)

Table S2 – Maternal and perinatal outcomes in eleven cases of portal hypertension in pregnancy. Abbreviations: CD: cesarean delivery; GA: gestational age; ICP: intrahepatic cholestasis of pregnancy; IoL: induction of labor; LCIV: left common iliac vein; NST: nonstress test; NICU: neonatal intensive care unit; NVD: normal vaginal delivery; PPROM: preterm premature rupture of membranes; RDS: respiratory distress syndrome; TIPS: transjugular intrahepatic portosystemic shunt
